# Primary mediastinal Ewing’s sarcoma presenting with sudden and severe chest pain: a case report

**DOI:** 10.3389/fonc.2023.1290603

**Published:** 2024-01-12

**Authors:** Chen Su, Xiaobo Zhu, Junjie Zhang

**Affiliations:** ^1^ Department of Cardiothoracic Surgery, Wujin Hospital Affiliated with Jiangsu University, Changzhou, China; ^2^ Department of Cardiothoracic Surgery, Wujin Clinical College of Xuzhou Medical University, Changzhou, China

**Keywords:** Ewing’s sarcoma, mediastinum, case report, thoracic cancer, chest pain

## Abstract

Ewing’s sarcoma, characterized by small round cell morphology, is a rare malignancy, with mediastinal Ewing’s sarcoma being even less common. This case describes a distinctive presentation of primary mediastinal Ewing’s sarcoma in a 32-year-old male presenting with sudden and severe chest pain. Initial evaluation excluded cardiac and pulmonary emergencies, revealing a posterior mediastinal mass through advanced imaging. The patient’s clinical symptoms significantly improved following the complete resection of the tumor via thoracoscopy. Subsequent analysis incorporating imaging, histological, immunohistochemical and genetic findings led to the conclusive diagnosis of primary mediastinal Ewing’s sarcoma.

## Introduction

Ewing’s sarcoma (EwS) is a rare and aggressive malignant neoplasm primarily affecting the bones and soft tissues, with a predilection for the long bones of extremities (1, 2). However, in exceedingly rare instances, this aggressive tumor can manifest in the mediastinum. Due to the restricted mediastinal space and the marked malignancy of the tumor, patients frequently exhibit a rapid onset of clinical symptoms. In this case report, we delineate a particularly rare and perplexing presentation of EwS originating in the posterior mediastinum and manifesting with acute chest pain. The patient underwent thoracoscopic surgical exploration and tumor resection. The postoperative pathological diagnosis confirmed the presence of primary EwS in the mediastinum.

## Case report

**Figure 1:**
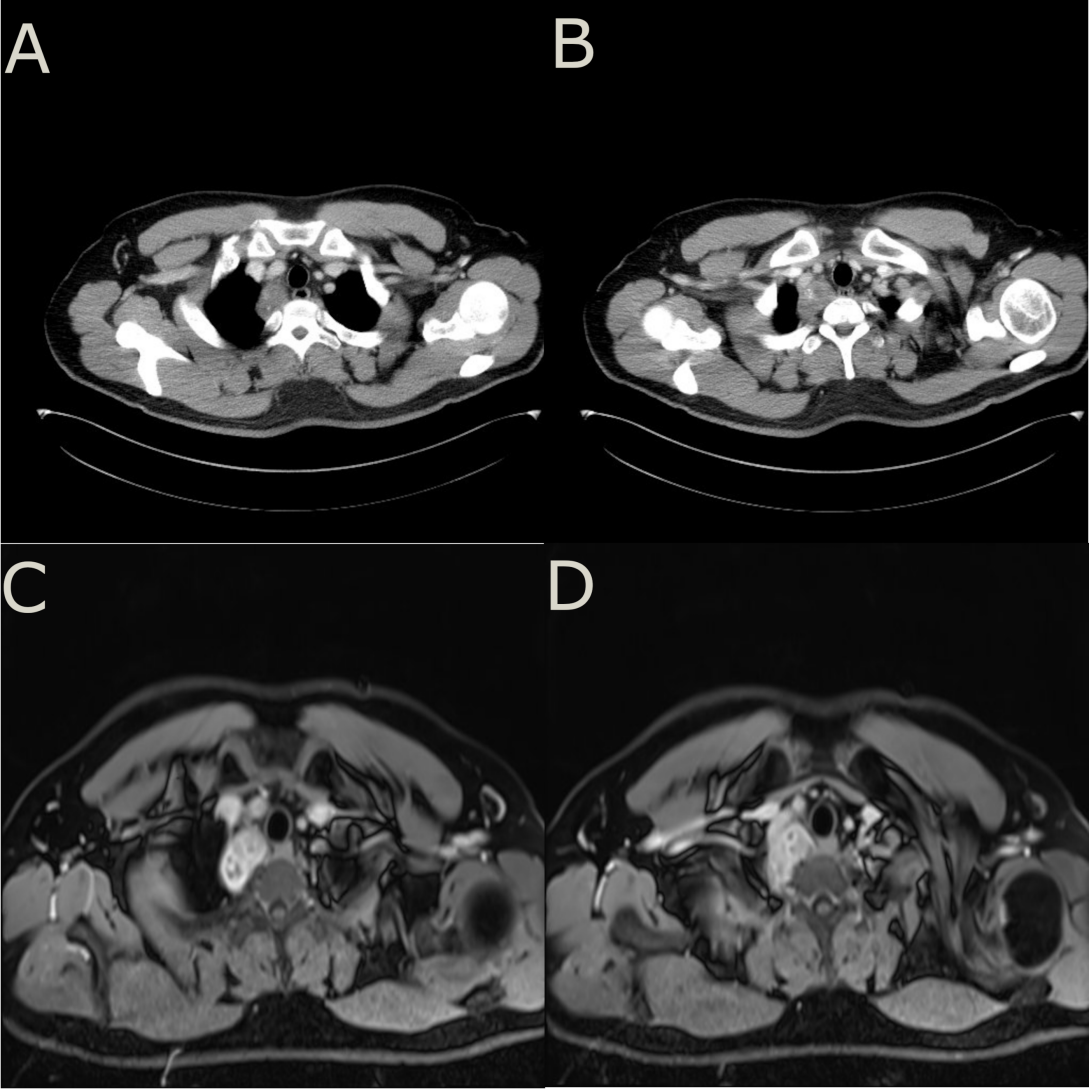
**(A, B)**, enhanced chest CT showed a mass adjacent to spine at the thoracic inlet with uneven density and the tumor is adjacent to 2nd and 3rd posterior ribs. **(C, D)**, enhanced MRI showed a higher signal intensity at the periphery of the tumor on T2-weighted images. The internal signal distribution was found to be heterogeneous, while the tumor margins were clearly demarcated with no apparent signs of adjacent tissue or structure infiltration.

**Figure 2:**
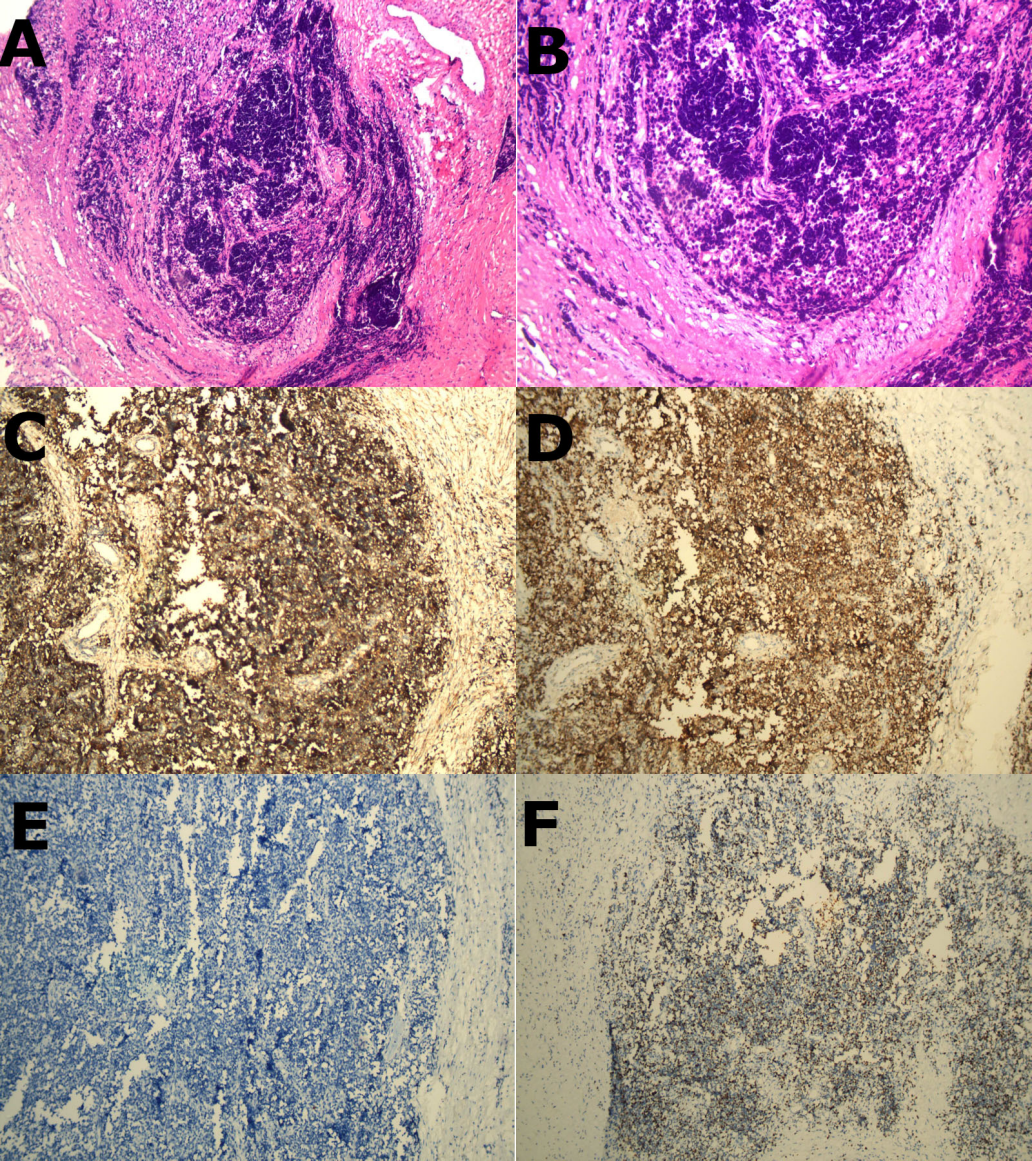
**(A, B)**, H&E staining revealed a homogeneous population of spherical cells with basophilic cytoplasm, and hyperchromatic nuclei (x100 and x200 respectively). **(C–F)** (immunohistochemistry), **(C)** showed strong positive expression of CD99 membrane staining. **(D)** showed strong positive expression of CyclinD1 membrane staining. **(E)** showed CK marking is negative in tumor cells. **(F)** showed the proportion of tumor cells exhibiting Ki-67 positivity was approximately 35%.

A 32-year-old young male patient presented to the emergency department with a 1-day history of severe right chest and back pain. He had no significant medical history and denied any tobacco or alcohol use. Initial vital signs at the emergency department showed a blood pressure of 150/90 mmHg, a heart rate of 100 beats/min, and an oxygen saturation of 98%. Cardiac markers and inflammatory markers were within normal limits, and the electrocardiogram (ECG) showed sinus rhythm without ST-segment elevation. Given the patient’s increasing discomfort, admission for further evaluation was arranged. Enhanced chest computed tomography (CT) revealed a 4cm x 4cm x 3cm mass in the right posterior lateral spine at the thoracic inlet, closely adjacent to the esophagus, trachea, right subclavian artery, right lung apex and posterior ribs. The tumor exhibited well-defined margins, heterogeneous internal density and partial internal enhancement after contrast enhancement (**Figures 1A, B**). In addition, laboratory testing showed normal tumor markers and lactate levels. Contrast-enhanced magnetic resonance imaging (MRI) revealed high signal intensity at the periphery and moderate signal within the interior on T2-weighted images (**Figures 1C, D**). Subsequent 18F-flurodeoxyglucose positron emission tomography/computed tomography scan showed no evidence of distant malignancy. The preliminary diagnosis was a primary mediastinal neurogenic tumor. After the failure of common analgesics to relieve the symptoms, the patient consent to surgery. Thoracoscopic exploration of the chest and tumor resection were performed on the fourth day after admission. Intraoperatively, the pleural cavity was free of adhesions and pleural effusion. The tumor, located in the right pleural apex, exhibited a smooth cystic wall with a rich vascular supply on its surface, and no evidence of invasion. The tumor was successfully excised *en bloc* via thoracoscopy, and subsequent histopathological analysis revealed a small round cell malignant tumor (**Figures 2A, B**). Immunohistochemical testing demonstrated strong positivity for CD99, CyclinD1, and negativity for CK, EMA, S-100, MyoD1, CD20, WT-1, Bcl-2, Vimentin and Desmin. The proliferation marker Ki-67 was found to be approximately 35% (**Figures 2C–F**). All indicators strongly suggest the presence of a sarcoma in this patient. To refine the classification of the specific sarcoma subtype, we engaged expert pathologists from Shanghai Zhongshan Hospital to reassess the histologic and immunohistochemical findings. Ultimately, genetic testing confirmed the existence of EWSR1::FLI1 fusion within the sarcoma tissue. Based on the combined findings of histopathological analysis, immunohistochemical and genetic testing, the conclusive diagnosis was recognized as primary mediastinal EwS. Postoperatively, the patient had no complications and was discharged. Continued chemotherapy was recommended, but due to financial constraints, he declined radiotherapy or chemotherapy during follow-up. Remarkably, he has survived nearly one year since the operation, attributing his longevity to his mindset. Despite recommendations for postoperative CT and chemotherapy, he remains resilient in his decision.

## Discussion

EwS is a highly aggressive and poorly prognostic malignant tumor that usually originates in the metaphysis of the long bones and the pelvis (3). It primarily affects children and young adults, with a slight male predominance (4). The EwS family includes extra-skeletal EwS, accounting for around 12% of EwS cases, and mainly occurs in the thigh, upper arm, and shoulder (5). However, EwS originating in the mediastinum is extremely rare, with no more than 20 cases of mediastinal EwS reported in the literature. Given its low incidence and atypical clinical manifestation, accurate diagnosis, differential diagnosis, and subsequent treatment options pose numerous challenges.

EwS displays a rapid growth pattern, and when growing in the mediastinum, it may compress the trachea, resulting in symptoms such as chest tightness, wheezing and coughing (6, 7). Furthermore, compression of the heart or major blood vessels can lead to mediastinal shift or superior vena cava obstruction syndrome, while intercostal nerve compression may cause chest pain (8, 9). These clinical manifestations of mediastinal EwS are atypical and may easily be mistaken for other mediastinal tumors or diseases. In this particular case, the patient was urgently admitted to the hospital due to severe chest pain accompanied by back pain. Given the patient’s age, potential conditions such as aortic dissection, pulmonary embolism, and tension pneumothorax could not be eliminated as possibilities. However, enhanced chest CT and ECG were able to rule out these acute and debilitating illnesses. Although the posterior mediastinal mass visible in preoperative imaging was initially deemed to be a neurogenic tumor, its location in the cervical pleura made it unsafe to perform a puncture biopsy for pathology. From these perspectives, the differential diagnosis of a mass found in the mediastinum is extensive and encompasses lymphomas, germ cell tumors, thymic tumors, and neurogenic tumors, necessitating a comprehensive approach to rule out various possibilities. CT and MRI play a crucial role in localizing and characterizing mediastinal tumors.

Histopathological evaluation, immunohistochemistry and genetic testing are all indispensable for confirming the diagnosis of primary mediastinal EwS. A prominent characteristic of EwS is the abundance of solid sheets of small round blue cells with high ratio of nuclear to cytoplasmic volume and scant eosinophilic cytoplasm, as observed in Hematoxylin and eosin (H&E) staining (7, 10, 11). It is crucial to acknowledge that other tumors might display comparable H&E staining characteristics, such as embryonal rhabdomyosarcoma, neuroblastoma and lymphoma (11). Therefore, the precise diagnosis of primary mediastinal EwS necessitates a range of techniques, including immunohistochemistry, genetic testing and other procedures. CD99, a protein product encoded by MIC2, serves as a crucial molecular marker for the diagnosis of EwS (1, 11). Although there are limited reports on primary mediastinal EwS cases, CD99 has been found to exhibit robust positive expression. However, the diagnostic specificity of CD99 for EwS is not 100%, and strong positive expression of CD99 can also be manifested in ependymoma (12). At the same time, the diagnostic sensitivity of CD99 for EwS is not 100%, and a small number of EwS cases can be weakly positive or negative for CD99 (13). NKX2.2 is considered to be another highly specific molecular marker in the diagnosis of EwS (14). Its combination with CD99 may improve the accuracy of diagnosis of EwS. Notably, histology and immunohistochemistry cannot definitively diagnose EwS, and further accurate diagnosis depends on genetic testing. Genetic testing can help rule out other types of sarcomas, including round cell sarcomas with EWSR1::non-ETS fusions, CIC-rearranged sarcomas, and sarcomas with BCOR gene alteration (15). Typically, FET::ETS gene family fusion can be used for the diagnosis of EwS (16). The EWSR1::FLI1 fusion formed by t(11,22)(q24;12) chromosomal translocation can be found in about 80% of EwS cases (13).

Due to its rarity, the optimal management of mediastinal EwS remains a subject of debate and a corresponding guideline reference is lacking. Current therapeutic strategies are predominantly adapted from the treatment regimens utilized for osseous EwS, encompassing a multimodal approach that includes surgery, chemotherapy, and radiotherapy. Surgical resection is considered a crucial intervention when viable, as complete tumor removal can alleviate symptoms and facilitate subsequent adjuvant therapies. ESMO Clinical Practice Guidelines suggest initiating combination chemotherapy for 3 to 6 cycles post-tumor diagnosis, followed by local treatment, and then up to another 6 to 10 cycles of chemotherapy. The duration of treatment is estimated at 10 to 12 months. The combination chemotherapy drugs encompass doxorubicin, cyclophosphamide, ifosfamide, vincristine, actinomycin, and etoposide (18). The EURO EWING 2012 phase 3 trial compared European and US chemotherapy regimens for newly diagnosed EwS. The study found that dose-intensive chemotherapy with vincristine, doxorubicin, cyclophosphamide, ifosfamide, and etoposide was more effective, less toxic, and shorter in duration, establishing it as the recommended standard of care for EwS (19). Radiotherapy is indicated in cases with incomplete excision, positive surgical margins, or inoperable tumors (20). The post-operative radiation treatment dose ranges from 45 to 60 Gy, contingent on tumor resection margin, reaction, and location (18). While multimodal therapy is the conventional method, tailored treatment plans should be taken into account to accommodate the distinct circumstances of each patient. When there is no evidence of local or distant metastases and complete removal is possible, surgery should be chosen without delay. We summarize most of the case studies on primary mediastinal EwS in **Table 1**. Tumor biopsy should be site-specific in the mediastinum, CT-guided transthoracic biopsy is preferred. In case of need, endobronchial ultrasound guided transbronchial needle aspiration or mediastinoscopic biopsy can be utilized. Occasionally, the tumor is extensive or infiltrates adjacent organs and tissues, and neoadjuvant therapy may provide patients with access to surgery. Multidisciplinary management emerges as the primary strategy to augment the survival rate of patients afflicted with mediastinal EwS. Beyond traditional surgical, radiotherapeutic, and chemotherapeutic interventions, molecular therapeutic strategies may be integrated clinically in potential future scenarios. Inhibitors of the histone demethylase LSD1 selectively induce programmed cell death in EwS cell lines and are presently under evaluation in a clinical trial (26). The recommended follow-up strategy is to undertake MRI examinations every 2 to 3 months post-treatment, to scrutinize the condition of the soft tissue within the mediastinum. If survival surpasses 5 years, a follow-up may be conducted biannually (18). Our patient’s one-year survival after surgery, despite the unavailability of comprehensive adjuvant therapies, suggests that surgery can play a crucial role in managing early mediastinal EwS. Early studies indicate that patients with EWSR1::FLI1 fusions have higher disease-free survival rates than patients with other types of fusions (17, 27). Nonetheless, it is important to acknowledge that our study is limited to a single case, and given the aggressive nature of EwS, it is imperative that eligible patients receive standard postoperative chemoradiotherapy to enhance their long-term survival. Further research and larger case series with more comprehensive follow-up data are warranted to establish the most effective treatment paradigm for this rare entity.

**Table 1:** Management and prognosis of primary mediastinal Ewing’s sarcoma in previous studies.

Study	Age and Sex	Site	Biopsy	Treatment	Prognosis
Reali A, et al (9)	66-F	Anterior mediastinum	Mediastinoscopy	Unresectable, chemotherapy and radiotherapy	Post-treatment, multiple pulmonary metastases appeared two months later
Koc U, et al (21)	63-M	Anterior mediastinum	NA	Unresectable, chemotherapy	NA
Cui M, et al (22)	66-M	Anterior mediastinum	None	Thoracotomy	Post-surgery, pericardial metastasis appeared two months later
Silver JM, et al (23)	17-M	Posterior mediastinum	None	Thoracotomy, postoperative chemotherapy and radiotherapy	NA
Caltavituro A, et al (24)	30-F	Anterior mediastinum	None	Thoracotomy, postoperative chemotherapy and stem cell transplantation	Post-surgery, local recurrence appeared a few months later. But the patient is still alive after adjuvant therapy
Halliday J, et al (7)	16-F	Anterior mediastinum	An open biopsy of the neck was performed but failed	Thoracotomy	NA
Halefoglu AM, et al (25)	19-F	Posterior mediastinum	CT-guided biopsy	Thoracotomy	NA
Ata F, et al (8)	16-M	Anterior and posterior mediastinum	CT-guided biopsy	None	NA
Li X, et al (6)	15-F	Anterior mediastinum	CT-guided biopsy	Chemotherapy	Remission

M, male; F, female; NA, not applicable.

## Conclusion

Our case illuminates the intricate diagnosis and management of primary mediastinal EwS, an uncommon and highly aggressive malignancy with an atypical presentation. We aim to expand the existing knowledge on this unusual entity and promote further research to enhance therapeutic strategies for primary mediastinal EwS. Furthermore, it underscores the indispensable significance of an interdisciplinary approach in handling rare and intricate cases in thoracic surgery and oncology.

## Data availability statement

The original contributions presented in the study are included in the article/supplementary material. Further inquiries can be directed to the corresponding author.

## Ethics statement

Ethical approval was not required for this case report as it involved standard clinical practice, maintained patient confidentiality, and obtained informed consent for data usage, aligning with established ethical principles. The studies were conducted in accordance with the local legislation and institutional requirements. Written informed consent was obtained from the patients/participants for the publication of this case report.

## Author contributions

CS: Writing – original draft. XZ: Writing – review & editing. JZ: Conceptualization, Funding acquisition, Supervision, Validation, Writing – review & editing.
